# The Role of the Tumor Stroma in Ovarian Cancer

**DOI:** 10.3389/fonc.2014.00104

**Published:** 2014-05-13

**Authors:** Ben Davidson, Claes G. Trope, Reuven Reich

**Affiliations:** ^1^Department of Pathology, Oslo University Hospital, Norwegian Radium Hospital, Oslo, Norway; ^2^University of Oslo, Faculty of Medicine, Institute of Clinical Medicine, Oslo, Norway; ^3^Department of Gynecologic Oncology, Oslo University Hospital, Norwegian Radium Hospital, Oslo, Norway; ^4^Institute of Drug Research, School of Pharmacy, Faculty of Medicine, The Hebrew University of Jerusalem, Jerusalem, Israel

**Keywords:** ovarian carcinoma, stromal myofibroblasts, metastasis, tumor progression, prognosis

## Abstract

The tumor microenvironment, consisting of stromal myofibroblasts, endothelial cells, and leukocytes, is growingly perceived to be a major contributor to the pathogenesis and disease progression in practically all cancer types. Stromal myofibroblasts produce angiogenic factors, proteases, growth factors, immune response-modulating proteins, anti-apoptotic proteins, and signaling molecules, and express surface receptors and respond to stimuli initiated in the tumor cells to establish a bi-directional communication network in the microenvironment to promote tumor cell invasion and metastasis. Many of these molecules are candidates for targeted therapy and the cancer stroma has been recently regarded as target for biological intervention. This review provides an overview of the biology and clinical role of the stroma in ovarian cancer.

## Introduction

Cancer is characterized by uncontrolled cell growth due to the combined effect of growth-promoting and cell death-suppressing signaling. Tumor growth and progression in carcinomas characteristically involves a pre-invasive phase, followed by invasion of the surrounding stroma, entry into blood and lymphatic vessels, and metastasis. It is growingly perceived that all these phases require cross-talk between tumor cells and their microenvironment, which consists of immune system effectors, endothelial cells, and stromal myofibroblasts. The latter cell population, often referred to as cancer-associated fibroblasts (CAF), has a particularly important role in tumor biology, due to its ability to dynamically modify the composition of the extracellular matrix (ECM), thereby facilitating invasion and subsequent metastatic colonization, and to produce and secrete tumor-promoting factors (1–3). This has impacted on the development of therapeutic strategies designed at targeting stromal myofibroblasts in cancer (4).

Ovarian cancer, the most lethal gynecologic malignancy (5), is a heterogeneous group of malignant tumors, of which ovarian carcinoma (OC) is the most common one. The common histological types of OC – serous, endometrioid, clear cell, and mucinous carcinoma, are distinct morphological entities that are growingly perceived to be of different etiology, with unique genetic and phenotypic characteristics and different clinical behavior, including response to chemotherapy (6). OC patients are diagnosed with advanced-stage disease in the majority of cases, and despite aggressive surgery combined with platinum-based chemotherapy often succumb to their disease, primarily due to chemoresistance in recurrent tumors (7).

As in other cancers, the OC stroma produces and expresses myriad molecules relevant for tumor biology, and the mere presence of a large stroma component in OC was reported to be associated with poor survival in advanced-stage disease (8). This review summarizes current data regarding the expression and clinical relevance of molecules related to the cancer microenvironment in OC stromal cells. Data related to the immune system or to the tumor vasculature are not discussed. Studies of areas which remain controversial, such as the role of mesenchymal stem cells in OC biology, are similarly not the focus of this paper.

## Proteases

Proteases are critical mediators of invasion and metastasis and are the cancer-associated molecules which have been most frequently studied in the OC stroma. Studies have predominantly focused on the matrix metalloproteinase (MMP) family, but a significant number of papers have focused on urinary-type plasminogen activator and cathepsin D.

Matrix metalloproteinases are a family of at least 23 membrane-bound (MT-MMP) or secreted zinc-dependent endopeptidases involved in invasion, tumor growth, inflammation, and angiogenesis. MMP family members share several domains, including a signal peptide required for secretion, a propeptide which keeps the enzyme latent, catalytic domain, and hemopexin-like domain, the latter required for binding tissue inhibitors of metalloproteinases (TIMP) and MMP activation. MMP-2 (Gelatinase A, 72 kDa type IV collagenase) and MMP-9 (Gelatinase B, 92 kDa type IV collagenase) additionally contain a collagen-binding area adjacent to their catalytic domain. In addition to ECM molecules, MMP substrates include proteases (other MMPs, plasminogen), growth factors (transforming growth factor; TGF), tyrosine kinase receptors (epidermal growth factor receptor, fibroblast growth factor receptor; EGFR, FGFR1), adhesion molecules (CD44, E-cadherin, αV integrin), chemokines, and the metastasis inhibitor KISS-1. MMPs are negatively regulated by various proteins, including TIMP-1–4, α2 macroglobulins, thrombospondins, and RECK. However, MMP-2 activation requires the formation of a complex with TIMP-2 and MT1-MMP (MMP-14) (9–11).

Collagen I and an anti-β1 integrin antibody induced activation of proMMP-2 in OC-derived fibroblasts *in vitro* (12). OC cell lines implanted in the peritoneal cavity of mice lacking the MMP-9 gene had fewer and smaller tumors than cells injected into mice with wild-type MMP-9 (13). MMP-2, MMP-9, MT1-MMP, and MT2-MMP were detected in the mouse stroma in animals inoculated with OC cells, but only MMP-2 and MT1-MMP levels were increased compared to normal mouse ovaries. Stromal expression of these molecules was unrelated to metastasis, the latter being rather related to tumor MT1-MMP levels (14).

The presence of stromal MMP-1, MMP-2, MMP-9, MT1-MMP, and TIMP-2 mRNA and/or protein has been shown in multiple studies of clinical OC specimens (15–35). However, the clinical significance of MMP and TIMP expression in the OC stroma remains controversial. In analysis of 90 primary OC, MMP-2, MMP-9, and MT1-MMP protein expression in stromal cells by immunohistochemistry (IHC) was significantly related to advanced-stage disease and poor disease-specific survival (DSS). Stromal MMP-9 and MT1-MMP were independent prognosticators in multivariate analysis (28). Higher stromal MMP-9 protein expression was similarly related to poor DSS in univariate, though not multivariate, analysis in another study (31). Stromal MMP-2 protein expression was related to shorter overall and disease-free survival (OS, DFS) in endometrioid, but not in serous OC in a third report (27). In contrast, in a smaller study of 33 OC, absence of MMP-2 from the OC stroma was associated with more aggressive disease (20). TIMP-2 mRNA expression in stromal cells of both primary OC and OC metastases was associated with poor outcome in univariate analysis, whereas the presence of MT1-MMP mRNA in stromal cells in metastases correlated with significantly longer survival. The association between stromal TIMP-2 mRNA expression in primary carcinomas and poor survival retained its significance in a multivariate analysis. Stromal MMP-2 and MMP-9 mRNA expression in primary or metastatic disease was unrelated to survival (19). In contrast, stromal TIMP-2 protein expression was significantly related to better chemoresponse and longer progression-free survival (PFS) and OS in analysis of 43 tumors (33).

Stromal expression of MMP-2 (30–32, 34), MMP-7 (34), MMP-9 (34), MMP-11 (32), MT1-MMP (34), TIMP-1 (34), and TIMP-2 (34) proteins was unrelated to survival in several studies.

The glycoprotein extracellular matrix metalloproteinase inducer (EMMPRIN; CD147) is member of the immunoglobulin superfamily of adhesion molecules, which stimulates the synthesis of several MMPs and binds MMP-1 and integrins on the surface of tumor cells.

Extracellular matrix metalloproteinase inducer was detected in tumor cells in primary OC, solid metastases, and malignant effusions in OC, as well as in stromal cells and endothelial cells. In solid lesions, EMMPRIN mRNA by *in situ* hybridization (ISH) was significantly co-expressed with β1 integrin mRNA in stromal cells. In survival analysis, EMMPRIN protein expression in stromal and endothelial cells of primary carcinomas correlated with poor survival (36).

Extracellular matrix metalloproteinase inducer protein expression by immunofluorescence was found in both tumor and stromal cells in a study of 120 primary OC and 40 intraperitoneal metastases. The monocarboxylate transporters MCT1 and MCT4, reported to be associated with EMMPRIN expression and drug resistance, were additionally detected in these specimens (37).

Urokinase-type plasminogen activator (uPA) is a serine protease that is synthesized as a latent pro-enzyme and activated by several proteases, including plasmin, cathepsins B and L, and kallikreins (KLKs). uPA and its homolog tissue-type PA (tPA) cleave plasminogen to plasmin, thereby mediating degradation of fibrin and other ECM proteins and the activation of several MMPs, as well as growth factors such as basic fibroblast growth factor (bFGF), insulin-like growth factor (IGF), and TGF-β. The uPA receptor uPAR additionally binds ECM proteins and integrins. The plasminogen activator inhibitors PAI1 and PAI2 and the plasmin inhibitor α2 antiplasmin negatively regulate this system (38, 39).

Analysis of uPA mRNA and protein expression in 57 ovarian tumors and 8 abdominal metastases showed expression of uPA mRNA in epithelial cells in benign and borderline tumors, whereas poorly differentiated primary OC and metastases of different histological grade had predominantly stromal expression. In contrast, uPA protein expression was seen in both compartments (40). In another paper by this group, increased expression of uPA, uPAR, and PAI1 mRNA was found in poorly differentiated primary OC with solid growth pattern and in metastases compared to cystic, better differentiated tumors (41). Protein expression of uPA and uPAR, as well as several MMP members, was frequently seen in the OC stroma in both primary carcinomas and metastases, though uPA and uPAR were absent in the stroma of well-differentiated tumors (42). In a murine OC model, uPAR^−/−^ mice lacking uPAR in host mesothelial cells had reduced tumor and ability to form peritoneal metastases, as well as reduced ascites formation and longer survival compared to uPAR^+/+^ mice. In clinical specimens, higher stromal uPAR protein expression was seen in OC compared to normal ovaries, with higher expression associated with higher histological grade (43).

The ETS family of transcription factors regulates the transcription of a large number of cancer-associated molecules, including uPA, uPAR, MMP-7, and MMP-9, as well as the apoptosis inhibitor Survivin, the tumor suppressor Maspin, the cell cycle protein p21/CIP1, and Slug, mediator of epithelial-to-mesenchymal transition (EMT), thereby affecting many cellular processes, including angiogenesis, invasion and metastasis, and cell survival (44).

**Figure 1:**
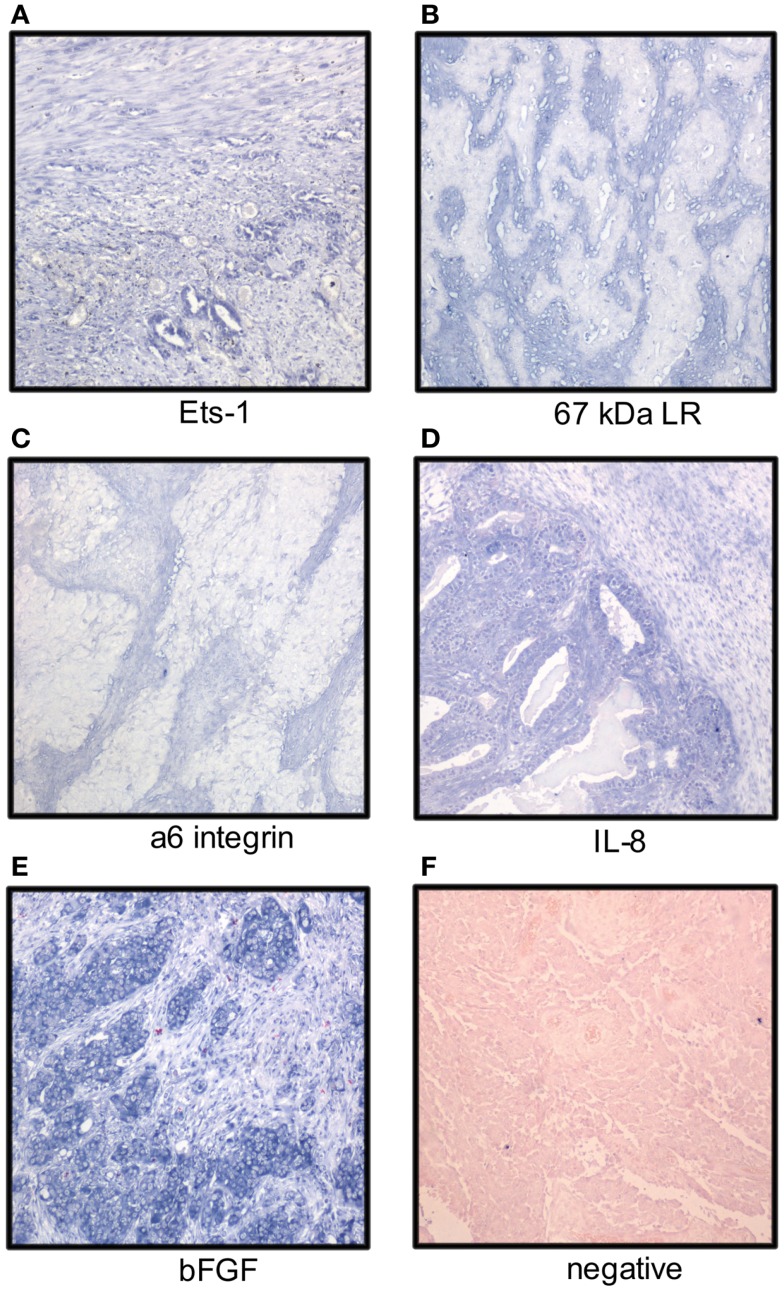
**Localization of mRNA of cancer-associated molecules to the ovarian carcinoma stroma**. OC stromal cells express mRNA of the Ets-1 transcription factor **(A)**, laminin receptors **(B,C)**, and the angiogenic factors IL-8 and bFGF **(D,E)**; **(F)** negative control. Tumor cells express Ets-1, IL-8, and bFGF (NBT-BCIP as chromogen, nuclear fast red as counterstain).

Ets-1 mRNA is co-expressed with MMP-1 and MMP-9 mRNA in the OC stroma (22). In analysis of 66 primary and metastatic OC from long-term and short-term survivors, Ets-1 mRNA was detected in stromal cells in 33% of cases using ISH (Figure 1), more often in tumors of short-term survivors, and was co-expressed with vascular endothelial growth factor (VEGF) mRNA. Ets-1 mRNA expression in both tumor and stromal cells was associated with poor survival in univariate analysis, and expression in stromal cells was an independent prognostic factor in a multivariate analysis (45).

In another study of the same cohort, the expression of PEA3, another Ets family member, was assessed using ISH. PEA3 mRNA was detected in stromal cells in 89% of tumors, but strong expression was limited to the stroma of grade 2–3 tumors. PEA3 mRNA expression in stromal cells was significantly related to MMP-2 mRNA expression in carcinoma cells, whereas PEA3 expression in carcinoma cells was significantly related to mRNA expression of the β1 integrin subunit, bFGF, and EMMPRIN in stromal cells. PEA3 mRNA was detected significantly more often in both carcinoma and stromal cells in tumors of short-term survivors and PEA3 expression in stromal cells correlated with shorter DFS and OS in univariate and multivariate survival analysis (46).

The clinical role of cathepsins, another family of proteases, was investigated in several studies. The level of cathepsin D, a lysosomal aspartyl protease, measured by immunoradiometric assay in OC tissue homogenates, was unrelated to clinical parameters or survival, with similar results for protein expression in tumor and stromal cells by IHC (47). In a study limited to stage III tumors (*n* = 185), tumor cell cathepsin D expression was related to longer OS in univariate analysis, with no such role for stromal expression. However, combined epithelial and stromal expression was an independent prognostic factor in multivariate analysis (48). No association was found with PFS. In contrast, cathepsin D expression in stromal cells was an independent prognostic factor of longer DFS, but not OS, in IHC analysis of 80 OC, with no prognostic role observed for tumor cell expression (49).

Cathepsin B, a cysteine protease, and the cysteine protease inhibitor cystatin C were detected in OC cells and their stroma, and were absent in cystadenomas (50).

Tissue KLKs are a family of 15 serine proteases encoded by a single gene cluster located at chromosome 19q13.4. Analysis of KLK4 expression in 43 primary and 63 metastatic OC showed stromal KLK4 expression in 48/103 specimens, which was significantly higher in primary tumors compared to metastases, with no prognostic role for this protein (51).

## ECM Proteins and Their Receptors

The ECM composition in OC and its clinical relevance has been the subject of several studies.

Analysis of mRNA expression of the proα1(I) and proα2(I) chains of type I procollagen and of the proα1(III) chain of type I procollagen by ISH demonstrated their localization to the OC stroma, whereas expression was weaker or absent in the stroma of benign cysts. In poorly differentiated carcinomas (*n* = 2), signals were additionally detected in tumor cells (52). Differences in the density of collagen type I fibers were observed between cystadenomas, borderline tumors, and OC of different histological grade in another study (53). Oncofetal fibronectin was detected in the OC stroma, but not in endometriosis, suggesting this protein was selectively expressed by the tumor microenvironment (54). Fibulin-1, an estrogen-regulated calcium-binding and acidic ECM glycoprotein, was localized to the OC stroma, with strongest expression in proximity to tumor cells, and its mRNA was localized to the latter compartment. Staining increased from normal ovaries through benign and borderline tumors to OC, and was associated with progesterone receptor, but not estrogen receptor expression (55).

Analysis of the expression pattern of laminin γ2 chain in mucinous ovarian tumors with gastrointestinal differentiation by IHC showed basement membrane localization in adenomas, borderline tumors, intraepithelial carcinomas, and adenocarcinomas with expansile growth pattern, whereas expression was cytoplasmic or stromal in carcinomas growing with infiltrative pattern (56). Stromal expression of laminin-5 γ2 chain with concomitant presence of MT1-MMP on the tumor cell surface was reported in clear cell OC (57). Galectin-1, a laminin-binding protein regulating tumor cell proliferation and adhesion to matrix, was overexpressed in OC compared to normal ovaries and co-localized with laminin-1 and fibronectin. Its levels were increased in fibroblasts cultured with OC cells *in vitro* with effect on tumor cell proliferation and adhesion (58). Analysis of the expression of two laminin receptors, the 67-kDa laminin receptor precursor (LBP) and the α6 integrin subunit, in 41 primary OC and 75 solid metastases showed mRNA expression by ISH in stromal cells in 68 and 20% of cases (Figure 1), respectively. No association with clinicopathologic parameters or outcome was found (59).

Analysis of additional integrin subunits in primary OC and solid metastases showed stromal expression of the β1 integrin subunit mRNA by ISH in 2 independent tumor series, whereas the αV subunit mRNA was found in the stroma in only one of the series. While tumor αV subunit mRNA expression was associated with poor survival in one of these studies, the presence of these subunits in stromal cells had no prognostic value (60, 61).

The mRNA expression of angiogenic cytokines and growth factors was analyzed in two studies. bFGF, interleukin-8 (IL-8), and VEGF mRNA was expressed in both tumor and stromal cells with no significant difference between primary carcinomas and metastases. bFGF was the most strongly and frequently expressed transcript in primary OC and in solid metastases in both series, with intermediate expression of IL-8 and low expression of VEGF (Figure 1). None of these factors was related to clinicopathologic parameters or disease outcome (62, 63). In another series, IL-8 mRNA expression was higher in tumor compared to stromal cells in OC specimens, whereas the protein was expressed in both compartments. IL-8 receptor B, but not A, was expressed in stromal cells (64). In a study of FGF-8 expression in OC, this cytokine was localized to tumor cells, whereas its receptors FGFR1, FGFR2, and FGFR4 were expressed by tumor cells, and to lesser extent, in stromal cells (65).

Hyaluronan (also termed hyaluronic acid or hyaluronate; HA), a large, linear, negatively charged polysaccharide with strong capacity to attract water, maintains tissue hydration and osmotic balance under normal condition. It additionally regulates cell adhesion, migration, apoptosis, and proliferation via interaction with specific cell surface receptors, which include the adhesion molecule CD44. HA has been shown to be involved in tumor progression of multiple cancers, through its effect on the above processes, as well as angiogenesis, invasion, and EMT (66).

HA is expressed in the stroma of both stage I and stage III OC, and its expression is increased in peritoneal metastases from patients with stage III disease compared to primary carcinomas (67). Analysis of 309 primary OC showed significant association between stromal HA expression and high histological grade, serous histology, advanced-stage and large residual disease volume, with no relationship to tumor cell CD44 expression. High stromal HA expression was further significantly related to poor relapse-free survival (RFS) and OS, and HA was more highly expressed in 45 patient-matched metastases additionally studied (68). Allelic imbalance at chromosome 3p21.3, a region harboring the hyaluronidase genes *HYAL1-3*, was found in microdissected tumor and stromal cells of borderline tumors and OC (69).

The unique stroma of clear cell OC was reported to contain both HA and collagen type IV, and these components were involved in its formation or modification (70, 71).

Proteoglycans, composed of a core protein to which glycosaminoglycan chains are attached, are a family of highly conserved macromolecules localized to the cellular membrane or the ECM. Proteoglycans are expressed by multiple cancers and mediate angiogenesis, tumor growth, invasion, and metastasis (72, 73).

Davies et al. analyzed the expression of syndecan-1–4, glypican-1, and perlecan in 147 ovarian specimens, including 115 OC, using IHC. Syndecan-1 was expressed in tumor and stromal cells of benign ovarian tumors, borderline tumors, and OC, with most intense staining in areas of invasion in OC, and was absent in normal ovaries. Syndecan-2 and -3 and glypican-1 were expressed in the stroma of all types of specimens, as was true for syndecan-4 in epithelial cells. Stromal perlecan expression was frequently seen in benign tissue and borderline tumors, but was lost in 67% of carcinomas. Stromal syndecan-1 expression was significantly associated with poor PFS and OS, though not independently (74).

In another study, stromal syndecan-1 and versican expression were associated with advanced-stage, serous histology, massive ascites, positive peritoneal cytology, and sub-optimal cytoreduction, as well as poor PFS and OS, though not independently (75). Ghosh et al. reported on overexpression of versican in OC compared to normal ovaries, as well as in advanced-stage compared to early-stage disease. Stromal versican expression was associated with higher microvessel counts, platinum resistance, and poor PFS and OS in univariate analysis (76). In another study, stromal versican expression was related to non-mucinous histology, advanced-stage, and reduced 5-year survival rate (77).

Decorin protein was reported to be expressed by the OC stroma, whereas tumor cells were negative, despite the presence of its mRNA in both cellular compartments (78). Periostin was overexpressed in the OC stroma compared to borderline and benign tumors and its presence in OC was associated with advanced-stage, disease recurrence, and poor OS, the latter also in multivariate analysis (79).

TGF-β is a ubiquitous cytokine with a dual role as both growth suppressor and promoter, effects which are largely mediated by the stroma and immune system. TGF-β acts predominantly as tumor promoter in several cancer types, including OC, and is consequently under consideration as a potential therapeutic target (80).

Comparative analysis of TGF-β1 and latent TGF-β1 binding protein 1 (LTBP-1) expression in serous and mucinous OC and adenomas showed strong stromal expression of these proteins limited to the former group (81). Transcriptome analysis of microdissected tumor and stromal cells from OC specimens and TGF-β-treated normal ovarian fibroblasts recently identified versican as an upregulated gene in CAF, and versican expression was upregulated by TGF-β, with resulting activation of the NF-κB signaling pathway and increased levels of CD44, MMP-9, and the hyaluronan-mediated motility receptor (82). Chloride intracellular channel 4 (CLIC4) was shown to mediate conversion of fibroblasts to myofibroblasts following stimulation with TGF-β1 *in vitro* and was frequently expressed in the OC stroma (83). Expression of TGF-β in the stroma of primary and recurrent OC was reported in another study (84).

Protein expression of the βA-subunit of activin A, member of the TGF-β superfamily, which regulates migration and invasion during EMT, metastasis, and MMP expression, was increased in stromal cells from OC specimens compared to adenomas (85).

Stromal protein and mRNA expression of secreted protein, acidic and rich in cysteine (SPARC; a.k.a osteonectin), a matricellular protein involved in angiogenesis and tumor invasion, was higher in OC compared to normal ovaries and borderline tumors. Tumor cells expressed SPARC protein, but not mRNA (86, 87).

Endothelins, mitogenic peptides with autocrine and paracrine effect, stimulated the growth of fibroblast cell lines isolated from ascites specimens of OC patients, and were found in both the tumor cell and stromal compartments in clinical specimens (88).

The platelet-derived growth factor receptors PDGFRα and PDGFRβ were expressed in stromal cells in 32 and 44% of OC in analysis of 170 tumors, but their expression was unrelated to clinical parameters or survival (89).

The granulin–epithelin precursor (GEP/progranulin/PC-cell-derived growth factor) is a 68-kDa secreted protein with several higher molecular weight forms due to glycosylation, most commonly of 88 kDa. GEP was shown to be a growth factor in OC (90). Analysis of 189 solid OC specimens (64 primary OC, 125 metastases) showed GEP expression in stromal and endothelial cells 52 and 67% specimens, respectively. Stromal GEP expression was significantly lower in metastases sampled during or following chemotherapy compared to chemo-naïve tumors, and the presence of GEP-positive stromal cells in untreated primary tumors correlated with worse OS (91).

Insulin-like growth factor-1 was detected in the OC stroma, with strongest expression around vessels, with less frequent and weaker expression in tumor cells (92).

## Transcriptional Regulators

HOX transcription factors constitute a large family of proteins that regulate embryogenesis and organogenesis via spatial cues, as well as by regulating apoptosis, proliferation, differentiation, motility, and angiogenesis. HOX members are differentially expressed in adult tissues and regulate the expression of cadherins, integrins, NCAM (CD56), and p53. Deregulation of HOX members has been shown in different cancers (93, 94).

HOXA7 was overexpressed in the tumor cell nuclei and in the stroma of clear cell OC compared to other OC histotypes, and expression was lowest in serous OC (95). HOXA9 expression in OC cells induced normal peritoneal fibroblasts and adipose tissue- and bone marrow-derived mesenchymal cells to develop CAF features, a process shown to be mediated by TGF-β2 upregulation of CXCL12, IL-6, and VEGF-A (96). HOXA10 expression in OSE cells stimulated interaction with the ECM proteins fibronectin and vitronectin, with omental mesothelial cells and fibroblasts (97).

DNA topoisomerase IIα (TOP2α), an enzyme involved in DNA replication, RNA transcription, chromosomal condensation, and mitotic chromatid separation, is the target of chemotherapeutic drugs such as etoposide and doxorubicin. Comparative analysis of primary and recurrent OC specimens showed reduced TOP2α expression in tumor cells in the latter group, whereas stromal expression was increased (98).

Vestigial like 3, a putative tumor suppressor, was expressed in high-grade serous OC cells, and to a lesser extent in stromal cells, in a series of 182 tumors, and higher stromal expression was associated with a trend for longer survival (99).

Nuclear expression of Snail1, one of the key regulators of EMT, was observed in tumor and stromal cells in 23 and 24% specimens, respectively, in a series of 74 OC. Snail1 expression was minimal in borderline tumors and absent in adenomas and normal ovaries. Snail1 tumor cell and stromal expression was unrelated to clinicopathologic parameters or survival (100).

Expression of two of four studied members of the CCAAT/enhancer binding protein (C/EBP) family of transcription factors, reported initially to regulate adipocyte proliferation and differentiation, was observed in the OC stroma, whereas all four proteins (C/EBP-α, -β, -δ, and -ζ) were expressed in tumor cells (101).

Nuclear expression of adrenal 4-binding protein/steroidogenic factor-1 (Ad4BP/SF-1) and dosage-sensitive sex reversal adrenal hypoplasia congenita critical region on the X chromosome gene 1 (DAX-1), nuclear receptor superfamily members involved in the regulation of steroidogenesis, was shown in stromal cells in OC. Enzymes involved in ovarian steroidogenesis, including steroidogenic acute regulatory protein (StAR), P450 side chain cleavage enzyme (P450scc), and 3-beta-hydroxysteroid dehydrogenase (3b-HSD) were detected in the stromal cell cytoplasm (102). Stromal protein expression of PPAR-β, another nuclear receptor superfamily member, was reduced in OC compared to borderline tumors, benign tumors, and normal ovaries, whereas expression of its target protein 3-phosphoinositide-dependent protein kinase 1 (PDK1) was limited to epithelial cells and increased in OC (103).

## Other Molecules

Various molecules related to other biological pathways have been localized to the OC stroma and are discussed in this section.

### Immune response effectors

Several studies have investigated the expression of molecules related to the immune response in OC stromal cells. Proteins reported to be expressed by stromal cells include IL-11 receptor (104), the pro-inflammatory peptide LL-37 and its precursor human cationic antimicrobial protein-18 [hCAP-18; (105)], lymphotoxin-β receptor and the chemokine CXCL11 (106), and CD277 (107), as well as IL-6, COX-2, and CXCL1 (108). The clinical role of these biomarkers in this cellular compartment remains to be established.

IL-1β was recently reported to suppress nuclear p53 expression in CAF. High IL-1β and its receptor IL-1R1 and low p53 expression in CAF were associated with poor OS. p53 knockdown in ovarian fibroblasts resulted in increased expression and secretion of IL-1β, IL-6, IL-8, VEGF, and growth-regulated oncogene-α (GRO-α) and increased tumor growth *in vivo* in a NF-κB-dependent manner (109). Induction of senescence in fibroblasts by GRO-α was previously reported to mediate tumor promotion in a previous study by the same group (110).

Ribonuclease-2 (RNASET2), an extracellular RNase expressed in the OC stroma, was shown to mediate recruitment of macrophages to the tumor microenvironment and its silencing enhanced tumor growth of OVCAR-3 cells *in vivo*. Genes altered following *RNASET2* silencing were involved in pathways related to the immune response and cell adhesion (111).

### Cell cycle and apoptosis-related proteins

Protein expression of the cell cycle inhibitor p16 in stromal cells was reported to be associated with improved prognosis, whereas the presence of this protein in tumor cells was a poor prognostic marker (112). Stromal expression of another cell cycle inhibitor, p27, was significantly reduced in OC compared to normal ovaries, as was the expression of lung resistance protein (LRP), a protein associated with multidrug resistance (MDR), whereas multidrug resistance protein (MRP) expression was not significantly different (113). Expression of tumor necrosis factor-related apoptosis-inducing ligand (TRAIL) and the death receptors DR4, DR5, and DcR1 was found in OC stromal cells (114). TRAIL was detected in the OC stroma in an additional study (115).

### Various molecules

The RNA-binding protein HuR and COX-2 were expressed in the OC stroma in 24 and 7% of specimens in a study of mucinous OC, with no clinical role observed for expression in this cellular compartment (116). Analysis of proteins related to the prostaglandin synthesis pathway using IHC showed expression of COX-2, microsomal prostaglandin E synthase-I (mPGES-I), and the prostaglandin E_2_ receptors EP_1_ and EP_2_ to the OC stroma, particularly in tumors of higher histological grade (117).

Expression of the α, β, and π sub-types of the detoxification enzyme glutathione *S*-transferase was observed in the stroma of OC specimens and different benign tumors (118).

Somatostatin and its receptors sst_1_, sst_2_, sst_3_, and sst_5_ were expressed with variable frequency in OC tumor cells and in their surrounding stroma, as well as in the stroma of different benign conditions. Somatostatin was significantly co-expressed with sst_1_, sst_2_, and sst_5_ in the stromal compartment in analysis of the entire cohort (119).

The serotonin receptors 5-HT1A, 5-HTA2, 5-HT2B, and 5-HT4 were expressed, to variable extent, in the stroma of normal ovaries, benign ovarian tumors, borderline tumors, and OC specimens, with 5-HT2B being the most expressed receptor (120).

Retinoic acid receptor-α was found in stromal fibroblasts, tumor-infiltrating lymphocytes, and OC cells in analysis of 16 tumors of serous or mixed histology (121).

Neural endopeptidase (CD10) was expressed in the stroma of serous borderline tumors and in OC of different histotype, whereas no staining was observed in mucinous borderline tumors, in benign tumors, and in normal ovaries (122).

Luteinizing hormone receptor mRNA expression analysis by RT-PCR and ISH was reduced in both tumor cells and the OC stroma compared to benign tumors, with intermediate levels for borderline tumors. Expression in grade 2–3 tumors was less frequent then in their grade 1 counterparts, and the receptor was absent in five analyzed metastases (123).

The expression of six different isozymes of aldehyde dehydrogenase, an enzyme implicated in stem cell biology in OC, was investigated in normal ovaries, adenomas, borderline tumors, and OC specimens. Stromal and tumor cell expression of several isozymes was found to differ between normal tissue and ovarian tumors, as well as between OC of different histotype (124).

Expression of class III β-tubulin was reduced, though not significantly, in the OC stroma following neoadjuvant chemotherapy in analysis of 22 paired tumors obtained pre- and post-chemotherapy. Tumor and stromal class III β-tubulin expression was associated with poor OS (125).

**Figure 2:**
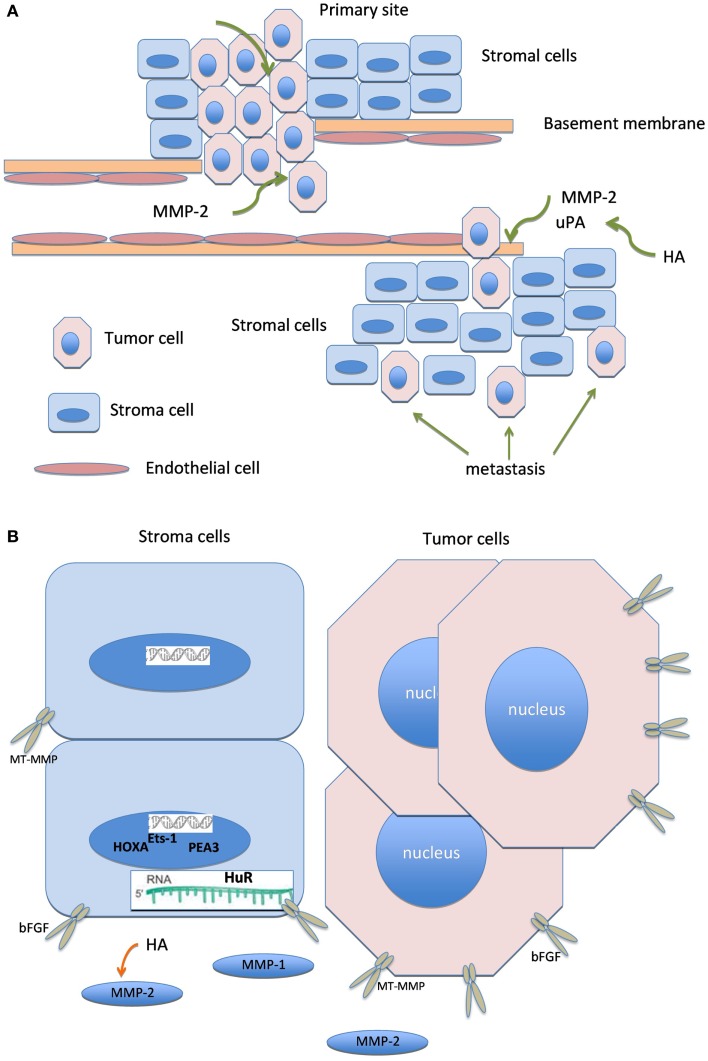
**Biologically linked cancer-associated molecules in ovarian carcinoma cells and the tumor stroma**. Graphical illustration linking molecules known to have biological association in this cancer, including hyaluronic acid (HA), basic fibroblast growth factor (bFGF), matrix metalloproteinases (MMP), urinary-type plasminogen activator, ETS transcription factors, HuR, and HOXA.

Graphical illustration linking molecules known to have biological association, including HA, bFGF, MMP members, uPA, ETS transcription factors, HuR, and HOXA is shown in Figure 2.

## Concluding Comments

Ovarian carcinoma is a highly lethal cancer characterized by considerable heterogeneity across different histological sub-types, as well as within the same morphological entity. In order to achieve noticeable improvement in the outcome of this disease, better understanding of the microenvironment of this tumor at both the primary site and metastatic locations is critically in need.

The above-discussed papers provide compelling evidence regarding the synthetic capacity of CAF in OC and emphasize the cross-talk between tumor cells and the stromal compartment; the latter interaction recently demonstrated *in vitro* (126). They additionally highlight the fact that the clinical relevance of a given molecule may be different or even opposite when expressed in carcinoma cells or in stromal cells. Nevertheless, many of these studies constitute single reports of the expression and clinical role of a given molecule, which need to be confirmed in series from other institutions, preferably studies in which each of the histological types of OC is studied separately.

Recent studies have applied high-throughput technology to the identification of central regulatory pathways in OC fibroblasts, often following microdissection, which allows for analyses focused on the target cell population. Qiu et al. studied genome-wide copy number and loss of heterozygosity (LOH) in CAF isolated from 25 OC and 10 breast carcinoma samples using SNP arrays. LOH and copy number alterations were rarely observed (127). Microarray analysis of microdissected stroma from 24 OC identified 52 candidate genes related to PFS, of which early growth response 1 (*EGR1*) and FBJ murine osteosarcoma viral oncogene homolog B (*FOSB*) were validated in an independent series of 50 tumors and found to be independent prognostic markers of poor PFS (128).

The role of miRNAs in reprograming of normal fibroblasts into CAF through downregulation of miR-31 and miR-214 and upregulation of miR-155 was recently shown, and the chemokine CCL5 was identified as target of miR-214, suggesting a role in modulation of the tumor microenvironment (129).

Exosomes are 30–100 nm lipoprotein vesicles containing proteins, mRNAs, and miRNAs that are secreted from cells and present in most circulating body fluids (130). Exosomes from SKOV-3 and OVCAR-3 cells induced adipose tissue-derived stem cells to acquire characteristics of myofibroblasts, with activation of the TGF-β pathway (131).

Lili et al. studied the stroma of 45 OC by microarray analysis and found two distinct signatures for the stromal compartment, characterized by different pairs of receptors and ligands (132).

Many of the molecules discussed in this review are expressed by both tumor and stromal cells and thereby present the possibility to target both cellular components in order to maximize the tumor-suppressive effect. While clinical studies aimed at inhibiting some of these cellular targets, e.g., proteases and COX-2, have been largely disappointing, other pathways, particularly receptor tyrosine kinase-driven pathways mediating angiogenesis and other tumor-related processes, are highly relevant (133, 134).

Therapeutic approaches are likely to focus to a larger extent on the tumor stroma in the future, as in the recent study by McCann and co-workers, in which inhibition of *Gli1*, part of the Hedgehog pathway, using the cyclopamine derivative IPI-926 in combination with chemotherapy was assessed (135). Whether such approaches could change the clinical course of OC is yet to be determined.

## Conflict of Interest Statement

The authors declare that the research was conducted in the absence of any commercial or financial relationships that could be construed as a potential conflict of interest.
